# Tracheotomy

**Published:** 1878-04

**Authors:** F. H. Blackman

**Affiliations:** Geneva


### TRACHEOTOMY.

By F. H. Blackman, M. D., of Geneva.

(*A Paper read before the Fox River Valley Medical Association, July* 2, 1877.)

*Case* 1.—C. P., aged four years, health always good. I saw him first on the evening of Jan. 29, 1877. His mother said he had not been well for two days, but had not thought him much sick. She said he was sick as his two little sisters had been, who were at this time convalescing. An examination of the fauces showed a small diphtheritic patch on one tonsil; externally on the same side the glands were slightly swollen and tender. I directed camphorated oil externally, and internally a saturated solution of chlorate of potass, alternated with quinine; and strong lemon juice applied to the fauces with a soft cotton-wool probang. I was to be notified if the boy was not better in the morning.

I heard nothing further of the case until Jan. 31, 11 p. m. Mr. P. came for me, saying he was afraid Charley had the croup. He was very much alarmed, as he had had two sons die from membranous croup, ten or twelve years before, and this was his only living son. I learned that the boy had been very much better since my former visit, and had only begun to develop symptoms of croup within the last three hours. He had been out to the barn several times during the day, which had been cold and rainy. I found him with considerable fever, fauces very red, no membrane except some slight strips far down in the pharyngeal space. He was very hoarse, and had considerable difficulty in breathing, and looked anxious and distressed. I gave an emetic of ipecac, which produced copious emesis; continued the chlorate of potass solution with compound syrup of squill added, and the quinine; the throat and upper part of the chest was covered with tobacco ointment and an onion poultice.

Feb. 1, 8 a. m.—The patient seemed much the same as on the night before. I applied a solution of nitrate of silver about the epiglottis, and commenced the use of lime water inhalations with the steam atomizer. 3 p. m.—Patient can only speak in a whisper, pulse 120, sweating, looks very tired, takes milk freely, some nausea. 11 p. m.—Voice extinct, breathing very labored. I catheterized the larynx and gave an emetic of the sulphate of copper, with some relief. I told the father that in my opinion tracheotomy was the only hope.

Feb. 2, 7 a. m.—Child still alive, but does not respond to the sulphate of copper, as formerly. 9 a. m.—Apparently dying. After a hurried preparation, with the assistance of the father, a one-armed man, I opened the trachea and introduced a tube extemporized from the silver catheter at hand. The operation was attended with very little hemorrhage. In about half an hour he could breathe without being prompted, and had taken a little milk and whisky, and in response to the question if he felt better, nodded affirmatively. The temperature of the room was maintained as near 80° as possible, and the air moistened with steam from the atomizer.

During the afternoon a double rubber canula was procured; just before which an accident happened which almost proved fatal, and had much to do with the result of the case. The tube becoming obstructed, and not succeeding immediately in removing the obstruction, the little fellow caught the tube and tore it out, causing considerable hemorrhage from the deep plexus of veins, he became asphyxiated from blood drawn into the trachea, and some little time elapsed before the bleeding could be stopped and respiration established.

Feb. 3, 8 a. m.—Had passed a good night; pulse 112; had taken milk frequently and with relish. The wound was dressed with weak carbolized oil, and the neck enveloped in flannel. The wound had the appearance of being covered with a thin film of false membrane, and the neck was a good deal swollen.

Feb. 3, 3 p.m.—Was summoned on account of difficult breathing, which cleansing the inside tube did not relieve; removed the entire canula and with a pair of forceps extracted a plug of fibrine, which was moving up and down in the trachea, and acting like a valve. There was a good deal of false membrane in the wound and extending down the trachea. The swelling of the neck had increased very much since morning. Auscultation discovered mucous râles. The breathing was relieved for a time, then went on increasing in difficulty, until about 8 p. m., when he suddenly died.

*Case* 2.—Elsie B., aged 4 years; previous health had been good, except occasional convulsions following whooping cough in her second year. I visited her first Jan. 28, 1877, for mild diphtheria.

Jan. 29.—Called and found Elsie improving; I did not see her again until Feb. 5, when I was called to see Fannie, another daughter, who was suffering from a malignant form of diphtheria, and who, by the way, had been taking sulpho-carbolate of soda since my former visit. Elsie was at this time running around the house apparently well, except an occasional very croupy cough. An examination revealed slight fever and a very reddened state of the fauces. The mother said she had been troubled with difficult breathing the two preceding nights, and she had herself administered ipecac. I prescribed bromide of potass and compound syrup of squill in a mixture, and recommended isolation from her sick sister.

Feb. 6, 9 a. m.—Patient evidently worse than on the previous day. The mother had been obliged to administer ipecac, which was followed by some relief and sleep.

Feb. 7, 9 a. m.—Patient had a very bad night; had been obliged to give much more ipecac to produce emesis than before; pulse 120; very great difficulty in breathing; voice almost extinct; still some appetite.

Feb. 7, 3 p. m.—Was summoned hurriedly by a servant, who said Elsie was dying. Found her struggling for breath, voice entirely extinct, lips and extremities blue. I gave an emetic of cupri sulphas, with full draughts of warm water, which produced copious emesis, followed by a hot foot-bath, hot compresses to the throat and chest, and the inhalation of lime-water vaporized by the steam atomizer, which relieved the more urgent symptoms. I proposed the operation of tracheotomy, and asked for assistance. 9 p. m.—Met Dr. C. N. Cooper, of Batavia, in council. The patient had had but one severe paroxism of dyspnœa since three o'clock. We remained with the patient until 1 a. m. Dr. Cooper went home to return again in the morning. I remained with the patient during the rest of the night.

Feb 8, 9 a. m.—Dr. Cooper having returned and death being imminent, I, with his and Dr. W. W. Ormsbee's assistance, the patient being etherized, opened the trachea below the isthmus of the thyroid gland. The loss of blood was trifling. The wound was thoroughly cauterized with solid stick nitrate of silver, and its angles brought together with strips of adhesive plaster. The neck was encircled with a strip of oiled silk, through a slit in which the tube was inserted into the trachea and held in place by tapes passing around the neck. A piece of old merino underwear, folded several thicknesses, was placed about the neck, a slit being cut for the inner tube. A small slit was cut in a second piece of oiled silk, and it was slipped over the inner tube to protect the merino from the sputa. A piece of wash blond lace was folded several times and laid over the open end of the tube, the air passing in and out through it, warming it, retaining, perhaps, some of the moisture of the exhaled air, and making it more natural. The expectoration was tinged with blood for an hour after the operation, when it became natural. The breathing was irregular for about the same length of time, probably from the combined influence of the dyspnœa and anæsthetic, after which the relief experienced from the operation was complete, respiration being 22, and the pulse 120 per minute. The air of the room was moistened by a large flat sponge filled with water and placed over the register of the furnace. The temperature was maintained at 76° or 78°.

Feb. 9th.—Removed the dressings and the entire tube. Cleansed the wound with a bit of sponge and warm water, and after drying, cauterized and redressed as before. On looking into the throat discovered diphtheritic patches on each tonsil; the nurse also called my attention to the vulva and anus where diphtheritic membrane existed. I cauterized them with nitrate of silver. The internal treatment was chlorate potass, and quinine with plenty of nourishing food. Complains of pain in her stomach probably due to the emetics.

Everything went on very favorably during the remainder of the treatment, the wound being dressed as above each day. I removed the tube the seventh day after the operation, fearing necrosis of the trachea, blood having appeared in the sputa, and although she could not breathe the natural way, the opening in the trachea remained open and breathing went on very well; on the second day after she began to expectorate and cough up the membrane, and fourteen days after the operation she could breathe very well but could not cry aloud. This continued about six weeks at the end of which time her recovery was complete.

My limited experience would hardly justify me in holding or in advocating very rigid opinions regarding this operation; still I have opinions somewhat as follows: Do not postpone the operation until the last stage, as I did in the foregoing cases, but operate early, as soon as the voice becomes a whisper and there is marked interference with oxygenation, especially if the child is under eight years of age.

I do no think the operation of itself is a source of much danger if certain precautions are observed. The too free use of emetics previous to this operation comes in for its full share in causing a fatal termination.

Operate under an anæsthetic if possible, and slowly, opening the trachea below the thyroid isthmus. Be sure and cauterize the wound with solid nitrate of silver at least once in croup, and in diphtheritic cases every day as long as the diphtheritic process is going on in the system. The wound should never be closed by stitches, approximation of its upper and lower angles by adhesive plaster is much preferable. Do not cauterize the inside of the trachea. Encircle the neck with either oiled silk or a rubber band with a slit in it for the tube; it is a protection to the wound and neck and prevents chafing. Old soft flannel about the neck is essential, but do not cover the end of the tube with it. Blond lace, washed to free it from starch, and folded several thicknesses is the best covering to respire through: several pieces can be prepared, dipped in some disinfectant and dried ready for use.

I prefer a hard rubber double canula having a uniform calibre, and of a size to fit the trachea, not too tightly or too loosely, as an ill fitting tube will cause necrosis of the trachea. Avoid drafts and maintain an uniform temperature. Dryness of the tube and air passages is best relieved by a few drops of tepid water. In order to be thoroughly prepared for this operation, the practitioner should have about five canulas sized as follows: one

25

a little under one-fourth inch in diameter for a child under three years; one one-fourth inch for a child between three and five years; one one-third inch for a child between five and eight; one two-fifths inch for those between eight and twelve, and one one-half inch for those between twelve and fifteen.

The smallest tube should be two and one-fourth inches in length and its curve should be one-fourth of a circle; the length should be increased one-fourth inch for each succeeding size named above. All should have the same curve.

I am satisfied that if tracheotomy was performed more frequently in this country, it would enjoy the popularity which it does in many parts of Europe.

